# Preoperative mitral valve annulus area size is an important factor in avoiding functional mitral stenosis after mitral valve repair

**DOI:** 10.1007/s12574-024-00671-x

**Published:** 2024-11-21

**Authors:** Haruka Sasaki, Hiroyuki Takaoka, Kazuki Yoshida, Moe Matsumoto, Yusei Nishikawa, Yoshitada Noguchi, Shuhei Aoki, Katsuya Suzuki, Satomi Yashima, Makiko Kinoshita, Noriko Suzuki-Eguchi, Shuichiro Takanashi, Goro Matsumiya, Yoshio Kobayashi

**Affiliations:** 1 Department of Cardiovascular Medicine, Chiba University Graduate School of Medicine, 1-8-1 Inohana Chuo-ku, Chiba-City, Chiba 260-8677 Japan; 2 Kawasaki Heart Center, Kawasaki Saiwai Hospital, Kawasaki, Japan; 3 Department of Cardiovascular Surgery, Chiba University Hospital, Chiba, Japan

**Keywords:** Degenerative mitral regurgitation, Mitral valve repair, Functional mitral stenosis, Three-dimensional transesophageal echocardiography

## Abstract

**Background:**

Functional mitral stenosis (FMS) following mitral valve (MV) repair for degenerative mitral regurgitation (DMR) is known as a poor prognostic factor. The parameters for avoiding postoperative FMS in MV repair for DMR have not been established.

**Methods:**

Two-hundred-and-twenty patients (mean age 61.1 ± 13.3 years, 144 males) who underwent MV repair for DMR were analyzed. MV annulus area was measured pre- and postoperatively using three-dimensional transesophageal echocardiography (TEE). Trans-mitral pressure gradient (TMPG) was evaluated by postoperative transthoracic echocardiography and FMS was defined as a mean TMPG ≥ 5 mmHg.

**Results:**

FMS was present in 14 patients (6.4%). Pre- versus postoperative MV annulus area change ratio was greater in the FMS group than in the non-FMS group (62.5 ± 7.2% vs. 48 ± 11.2%, *p* < 0.0001). On multivariate logistic regression analysis, MV annulus area change ratio was an independent predictor of FMS (odds ratio 1.19, 95% confidence interval 1.09–1.33, *p* < 0.0001), while receiver operating characteristics analysis showed that the optimal threshold for MV annulus area change ratio to predict FMS was 56.2% (area under the curve, 0.87; *p* < 0.0001).

**Conclusion:**

The preoperative MV annulus area on TEE can be used to determine the postoperative MV annulus area to avoid FMS after MV repair.

## Introduction

Mitral valve (MV) repair is currently the standard treatment for degenerative mitral regurgitation (DMR) [1, 2]. While good long-term clinical outcomes after MV repair have been reported, functional mitral stenosis (FMS) after MV repair for DMR is recognized as a poor prognosis factor [3]. Although the use of prosthetic valve rings in MV repair has been established as a standard technique, the evidence to determine the optimal size of the prosthetic valve ring in each case has not been available, and the choice is left to the surgeon’s experience [4]. A previous study reported that annuloplasty using a small-sized prosthetic valve ring in MV repair causes postoperative FMS [5], which suggests that the postoperative prosthetic ring area affects postoperative FMS. However, the most frequently used indicators for ring selection in MV repair such as anterior leaflet length and inter-trigone distance, reflect only one part of the valve, and to our knowledge MV annulus area has not been previously used.

Here, using transesophageal echocardiography (TEE), which allows detailed three-dimensional (3D) morphological evaluation and accurate measurement of the MV annulus, we evaluated pre- and post-operative MV annulus area and investigated its effect on postoperative FMS using TEE.

## Methods

### Study population

We retrospectively studied 240 consecutive patients who underwent a primary MV repair at Chiba University Hospital or Kawasaki Saiwai Hospital between April 2018 and December 2022. We excluded six patients whose left ventricular ejection fraction (LVEF) was less than 55%, eight patients whose postoperative TEE images were poor and whose MV annulus morphology was challenging to analyze, three patients with tachyarrhythmia at the time of postoperative transthoracic echocardiography (TTE), and three patients who underwent MV repair using autologous pericardium. Finally, 220 patients were available for analysis. Preoperative TTE, TEE, and intraoperative TEE were performed in all patients. Before discharge, the patients were evaluated for FMS by postoperative TTE. We defined FMS as a mean trans-mitral pressure gradient (TMPG) of 5 or higher with postoperative TTE based on previous reports [3, 6, 7]. The postoperative TEE was performed to evaluate postoperative MV annulus morphology for 146 patients before discharge; the remaining 74 patients were assessed for postoperative MV annulus morphology using intraoperative TEE images. We compared patient characteristics, echocardiographic characteristics, and procedure techniques between the groups with and without FMS. This study was approved by the institutional review board of Chiba University Graduate School of Medicine.

### Transthoracic echocardiography

TTE was performed pre- and postoperatively as routine clinical practice using the EPIQ system and X5-1 transducers (Philips Medical Systems, Andover, MA, USA) or Vivid E9 and M5S transducers (GE Vingmed, Horten, Norway) by standard methods according to the guidelines of the American Society of Echocardiography and the European Association of Cardiovascular Imaging [8]. The left atrial diameter (LAD), left ventricular end-diastolic diameter, and end-systolic diameters were measured in the parasternal long-axis view. LVEF was measured by the modified Simpson method in the apical view. The stroke volume (SV) was calculated by the pulse wave Doppler method as LV outflow tract (LVOT) area x LVOT velocity time integral (VTI). Trans-mitral pressure gradient (TMPG) was measured from the trans-mitral velocity flow curve using continuous wave Doppler. The postoperative MV effective orifice area (EOA) was calculated with the continuity equation using left ventricular SV and MV VTI.

### Transesophageal echocardiography

**Fig. 1:**
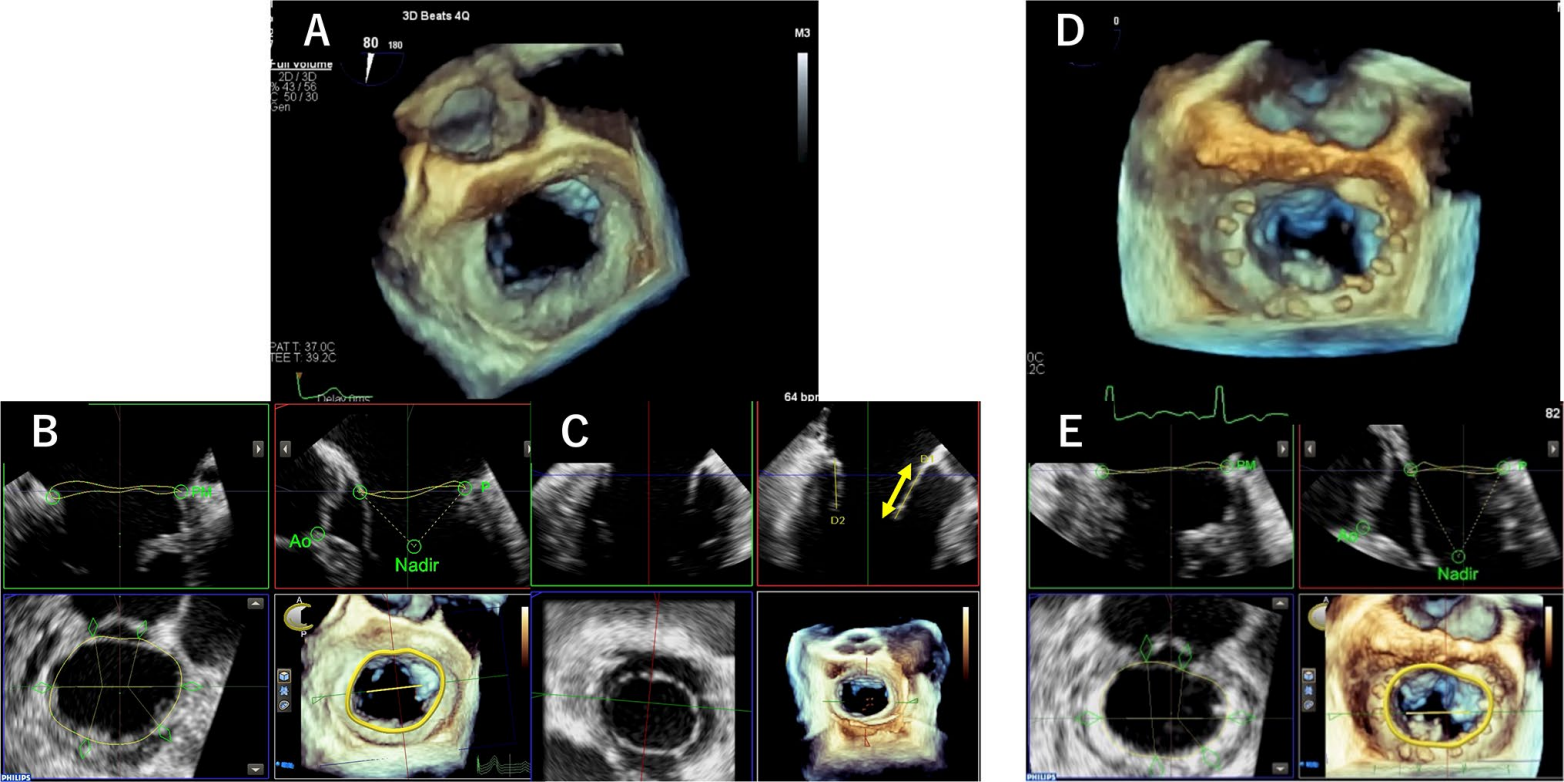
Methods of three-dimensional analysis of mitral valve using transesophageal echocardiography. From preoperative three-dimensional transesophageal echocardiographic (3D TEE) data (**A**), the mitral annulus area, circumference diameter (yellow circle), lateral diameter (pink arrow), and anterior to posterior diameter (blue arrow) of the mitral annulus were measured using MVN software at mid-diastolic phase (**B**). A two-dimensional long-axis view perpendicular to the anterior (A2)–posterior (P2) coaptation line was reconstructed, and the lengths of A2 (white arrow) were measured (**C**). From postoperative 3D TEE data (**D**), the mitral annulus area was measured using MVN software as well as preoperative analysis at the mid-diastolic phase (**E**)

TEE was performed using the EPIQ system with an X7-2t or X8-2t transducer (Philips Medical Systems, Andover, MA, USA). We obtained 3D full-volume MV data during breath-holding for six heartbeat compositions or four heartbeats in atrial fibrillation patients. The view was optimized for depth and gain setting before 3D acquisition for high spatial and temporal resolution data. For preoperative 3D data, we performed annulus analysis using MVN software (Philips Medical Systems, Andover, MA, USA). We measured MV annulus area, lateral diameter, anterior to posterior diameter, and aspect ratio in the mid-diastolic phase (Fig. 1A). The aspect ratio was calculated using the equation (anterior to posterior diameter/lateral diameter × 100). MV annulus area change ratio between pre- and post-MV repair (%) was calculated using the Equation (100 – [post-MV annulus area/ pre-MV annulus area] × 100). In addition, we acquired multiplanar reconstructed views perpendicular to the anterior (A2)–posterior (P2) coaptation line in the mid-diastolic phase and measured the anterior (A2) leaflet lengths (Fig. 1B). In the intraoperative and postoperative transesophageal echocardiography, 3D images of the MV were acquired in the same way as the preoperative data, and the MV annulus area was measured using MVN software (Fig. 1C). The echocardiographic images were transferred to and stored in a computer for offline analysis using IntelliSpace Cardiovascular (Philips Medical Systems, Andover, MA, USA).

### Operative procedure

MV repair in this study was performed via a median sternotomy approach (*n* = 196, 88%) or a minimally invasive cardiac surgery approach (*n* = 26, 12%). Posterior leaflet lesion and commissure leaflet prolapse were repaired by leaflet resection, neo-chordal placement, or both. Anterior leaflet prolapse was repaired by neo-chordal placement. Edge-to-edge repair was added when needed. The surgeon in charge of each patient determined the range and shape of the resection. Ring annuloplasty was performed in all cases. The type and size of the ring were chosen based on the surgeon’s preference according to MV morphology, and the ring size was selected based on the inter-trigonal distance or anterior leaflet size measured with the sizer provided.

### Statistical analysis

Continuous variables are expressed as mean ± standard deviation and categorical variables are summarized as percentages and counts. Continuous variables between patient groups were compared using the Student *t* test. The differences in proportions were examined by the *χ*^2^ test (Fisher’s exact test was applied when the number of observations was small). A *p* value less than 0.05 was considered significant. The correlation between TMPG and postoperative MV annulus area and area change ratio between pre- and post-MV repair were assessed using Spearman’s correlation coefficient. The associated variables in univariable analyses (*p* < 0.05) were included in the multivariable logistic regression analysis model to identify predictors of FMS after MV repair. The optimal cutoff values of the prediction of FMS were obtained from receiver operating characteristic (ROC) curve analysis, and areas under the curve were calculated. All statistical analyses were performed using the JMP software program, version 16.0.0 (SAS Institute Inc, Cary, NC, USA).

This study was approved by Chiba University Hospital and Kawasaki Saiwai Hospital Ethics Committee (Reference no. HK202307-04).

## Results

**Fig. 2:**
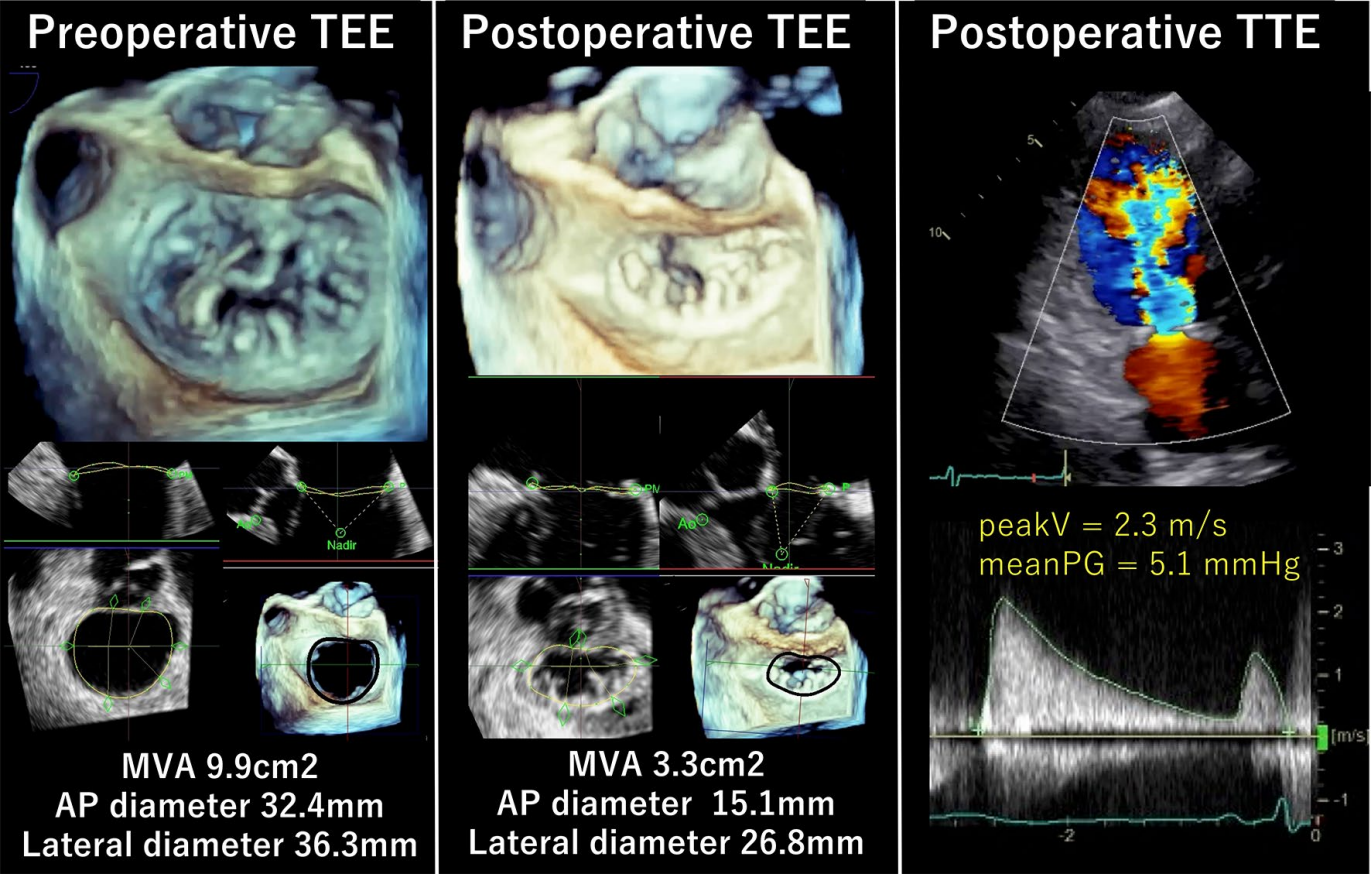
A representative case of postoperative functional mitral stenosis. A case of a 68-year-old female with P1 prolapse. The mitral valve annulus (MVA) was reduced from 9.9 cm^2^ to 3.3 cm^2^, and the area change ratio between pre- and post-MV repair was 67%. TEE; transesophageal echocardiography, TTE; transthoracic echocardiography, AP diameter; anterior to posterior diameter

A total of 14 patients (6.4%) were determined to have FMS after MV repair for DMR (Fig. 2). The comparison of clinical characteristics in patients with and without FMS showed no significant differences between the two groups (Table 1).

**Table 1:** Comparison of patient characteristics between patients with and without FMS

	FMS group (*n* = 14)	Without FMS group (*n* = 206)	*p* value
Age (years)	59.4 ± 14.2	61.2 ± 13.3	0.61
Men	7 (50)	142 (68.9)	0.24
Body surface area (m^2^)	1.6 ± 0.1	1.6 ± 0.2	0.81
Preoperative NYHA			0.49
I	3 (21.4)	86 (41.8)	
II	7 (50)	88 (42.7)	
III	4 (28.6)	26 (12.6)	
IV	0 (0)	6 (2.9)	
Hypertension	10 (71.4)	118 (57.3)	0.45
Diabetes mellitus	1 (7.1)	18 (8.7)	0.77
Chronic kidney disease	2 (14.3)	49 (23.8)	0.63
Atrial fibrillation	4 (28.6)	46 (22.3)	0.83
Coronary artery disease	1 (7.1)	29 (14.1)	0.74

*NYHA* New York Heart Association

### Preoperative echocardiographic parameters

Preoperative TTE and TEE parameters in patients with and without FMS are compared in Table 2. Preoperative LA and LV size, LV function, prolapse site, and MV morphological parameters in the two groups were similar. Prolapse sites were determined using TTE and TEE. The bi-leaflets lesions contained 13 cases of Balow’s type region and 5 cases of multiple lesions, including anterior and posterior segment, such as P1 and A3 prolapse or A2, A3, P2, and P3 prolapse.

**Table 2:** Comparison of parameters of preoperative transthoracic echocardiography and transesophageal echocardiography between patients with and without FMS

	FMS group (*n* = 14)	Without FMS group (*n* = 206)	*p* value
Transthoracic echocardiography			
LAD (mm)	48 ± 4.8	44.9 ± 7.1	0.11
LVEDD (mm)	53.3 ± 5.3	53 ± 7.6	0.87
LVESD (mm)	35 ± 5.5	33 ± 4.6	0.11
LVEF (%)	65.4 ± 4.5	66.9 ± 5.6	0.32
Prolapse site			0.60
Anterior prolapse only	5 (35.7)	37 (18)	
Posterior prolapse only	7 (50)	128 (62.1)	
Bileaflet prolapse	0 (0)	18 (8.7)	
Commissure prolapse	2 (14.3)	23 (11.2)	
Transesophageal echocardiography			
MV annulus area (cm^2^)	11.8 ± 4.1	11.3 ± 2.5	0.45
Circumference diameter of MV annulus (mm)	124.8 ± 21.1	123.9 ± 14.6	0.84
Lateral diameter of MV annulus (cm)	40.7 ± 7.1	39.9 ± 4.2	0.53
Anterior to posterior diameter of MV annulus (cm)	35.4 ± 6.3	34.6 ± 4.5	0.52
Aspect ratio of MV annulus (%)	87.4 ± 7.7	87 ± 9.2	0.86
Anterior leaflet (A2) length (mm)	24.4 ± 3.9	23.8 ± 3.0	0.49

*LAD* left atrial diameter, *LVEDD* left ventricular end-diastolic diameter, *LVESD* left ventricular end-systolic diameter, *LVEF* left ventricular ejection fraction, *MV* mitral valve, *FMS* functional mitral stenosis

### Types of implanted prosthetic valve ring

A full ring type was used for 105 (48%) patients, of whom 84 received the semi-rigid type (77 Physio II (Edwards Lifesciences, Irvine, CA, USA), 1 Memo 3D (LivaNova, Saluggia, Italy), and 6 SimuForm (Medtronic Inc., Minneapolis, MN, USA) rings) and 21 received the flexible type (21 Tailor (Abbott Laboratories, Abbott Park, IL, USA) rings). A partial band type was used for 115 (52%) patients, of whom 80 received the semi-rigid type (72 Physio Flex (Edwards Lifesciences, Irvine, CA, USA) and 8 Cosgrove (Edwards Lifesciences, Irvine, CA, USA) bands) and 35 received the flexible type (35 Tailor (Abbott Laboratories, Abbott Park, IL, USA) bands). The mean, minimum, and maximum postoperative MVA measured with postoperative TEE are summarized in Table 3 for each ring type and size in the main rings used (Physio II, Physio Flex, Tailor ring, and Tailor band). The full rings showed less variation in values for each ring size than partial rings. There were differences among products in the relationship between ring size and postoperative MVA.

**Table 3:** Postoperative mitral valve annulus area for the ring types and sizes

Full ring								
Physio2								
Ring size (mm)	26	28	30	32	34	36	38	40
MVA (cm^2^)	-	3.9 [3.8–4.0]	4.5 [3.4–4.5]	5.2 [5.2–5.6]	6.0 [5.9–6.1]	6.9 [6.8–6.9]	7.9	8.8
Number of cases	0	19	21	21	9	5	1	1

Tailor								
Ring size (mm)	27	29	31	33	35			
MVA (cm^2^)	4.7	5.6 [5.6–5.7]	6.4 [6.1–6.5]	7.3 [7.3–7.5]	8.1 [7.9–8.2]			
Number of cases	1	2	4	5	9			

Partial band								
Physio Flex								
Ring size (mm)	26	28	30	32	34	36	38	40
MVA (cm^2^)	3.9	4.2 [3.8–5.2]	5.2 [4.3–5.4]	5.8 [5.6–6.0]	6.4 [6.2–6.9]	7.8 [7.1–8.4]	8.3 [7.6–9.4]	9.8
Number of cases	1	5	15	19	18	10	3	1

Tailor band								
Ring size (mm)	27	29	31	33	35			
MVA (cm^2^)	4.6 [4.4–4.8]	5.1 [4.4–5.8]	6.5 [5.6–8.1]	6.9 [6.4–7.8]	8.3 [8.0–9.3]			
Number of cases	2	4	8	14	7			

*MVA*: postoperative mitral valve annulus area measured with postoperative transesophageal echocardiography. The average MVA values were listed with minimum and maximum values in brackets

### Surgical procedures

Surgical procedures in patients with and without FMS are compared in Table 4. A full ring-type prosthetic valve ring was used significantly more frequently in the group with FMS than in the group without. There was no significant difference between the two groups about the technique used for the valve leaflets.

**Table 4:** Comparison of surgical procedures between patients with and without FMS

	FMS group (*n* = 14)	Without FMS group (*n* = 206)	p value
Minimally invasive approach	4 (28.6)	22 (10.7)	0.14
Cardiopulmonary bypass time (min)	187 ± 64	198 ± 51	0.56
Aorta cross clamp time (min)	144 ± 39	130 ± 47	0.28
Repair technique			
Resection and suture	7 (50)	146 (70.9)	0.18
Chordal replacement	9 (64.3)	159 (77.2)	0.44
Edge-to-edge repair	1 (7.1)	4 (1.9)	0.70
Prosthetic valve ring			
Full ring	11 (78.6)	94 (45.6)	0.03
Semi-rigid type	13 (92.9)	151 (73.3)	0.19
Concomitant procedure			
Maze	4 (28.6)	31 (15)	0.34
Tricuspid annuloplasty	6 (42.9)	54 (26.2)	0.30
Coronary artery bypass grafting	1 (7.1)	28 (13.6)	0.78

*FMS* functional mitral stenosis

### Postoperative echocardiographic parameters

Parameters on postoperative TTE and TEE in patients with and without FMS are compared in Table 5. Postoperative TTE was performed an average of 6.2 ± 2.6 days after surgery. Mean TMPG was 5.4 ± 0.4 mmHg in the group with FMS and 2.4 ± 0.8 mmHg in the group without FMS. EOA calculated from the continuous equation using TTE was 1.7 ± 0.3 cm^2^ in the group with FMS and 2.3 ± 0.5 cm^2^ in the group without FMS. Postoperative MV annulus area on TEE was significantly smaller in the group with FMS than in the group without FMS (4.6 ± 1.0 cm^2^ vs. 6.0 ± 1.4 cm^2^, *p* = 0.001). The MV annulus area change ratio between pre- and post-MV repair was significantly larger in the group with FMS than in the group without FMS (62.5 ± 7.2% vs. 48 ± 11.2%, *p* < 0.0001).

**Table 5:** Comparison of parameters of postoperative transthoracic echocardiography and transesophageal echocardiography between patients with and without FMS

	FMS group (*n* = 14)	Without FMS group (*n* = 206)	*p* value
Transthoracic echocardiography			
LAD (mm)	40.5 ± 6.1	40.1 ± 25	0.95
LVEDD (mm)	47.8 ± 5.0	46.2 ± 6.2	0.34
LVESD (mm)	31.2 ± 5.3	32.7 ± 4.0	0.30
LVEF (%)	61.2 ± 5.4	59.9 ± 7	0.98
Stroke volume (ml)	61.9 ± 15.5	61.1 ± 17.3	0.86
Mean TMPG (mmHg)	5.4 ± 0.5	2.4 ± 0.8	< 0.001
EOA (cm^2^)	1.7 ± 0.3	2.3 ± 0.5	0.001
Residual mitral regurgitation			0.89
None	6 (42.9)	100 (58.5)	
Mild	8 (57.1)	106 (51.5)	
Moderate or above	0	0	
Transesophageal echocardiography			
MV annulus area (cm^2^)	4.6 ± 1.0	6.0 ± 1.4	0.001
MV annulus area/ BSA (cm^2^/ m^2^)	2.9 ± 5.4	3.6 ± 8.2	0.001
MV annulus area change ratio between pre- and post-MV repair (%)	59.1 ± 7.8	46.8 ± 11	< 0.001

*LAD* left atrial diameter, *LVEDD* left ventricular end-diastolic diameter, *LVESD* left ventricular end-systolic diameter, *LVEF* left ventricular ejection fraction, *TMPG* transmitral pressure gradient, *EOA* effective orifice area, *MV* mitral valve, *FMS* functional mitral stenosis

### Logistic regression analysis for prediction of FMS

**Fig. 3:**
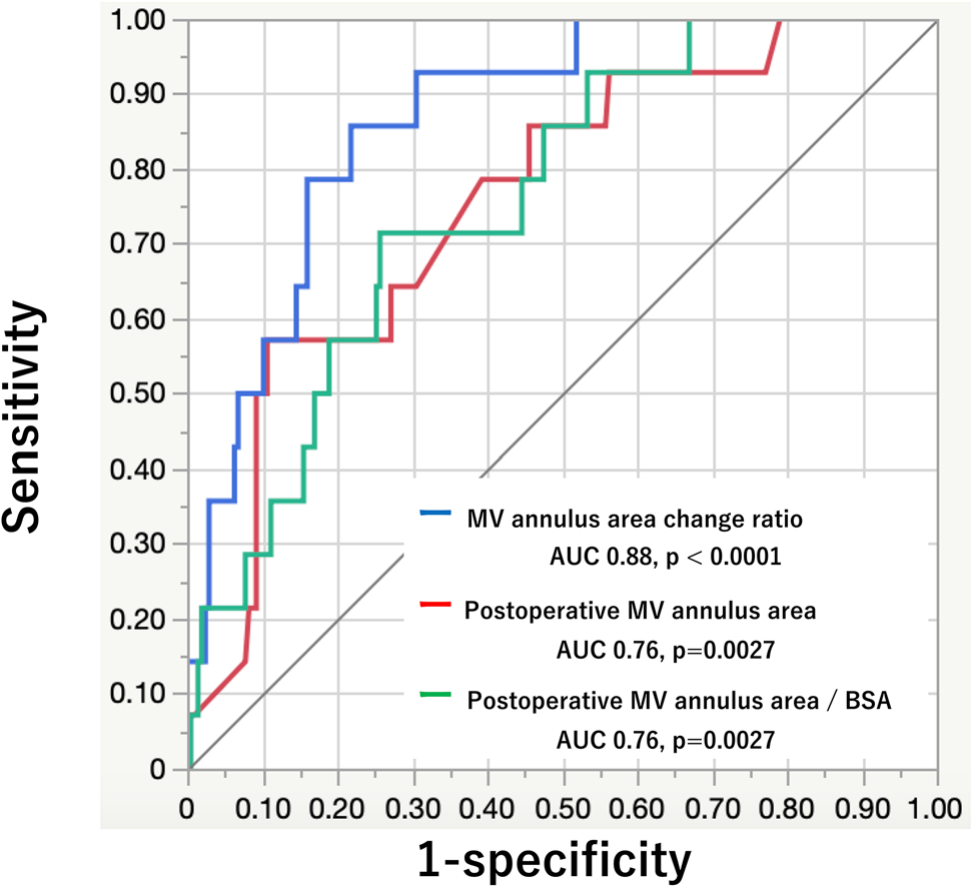
Receiver operating characteristic curve analyses of the mitral valve annulus area change ratio between pre- and post-mitral valve repair to predict postoperative functional mitral stenosis. The receiver operating characteristic curve demonstrates good discriminatory ability of the mitral valve annulus area change ratio between pre- and post-MV repair in predicting post-operative functional mitral stenosis (area under the curve (AUC) = 0.88, *p* < 0.0001). A mitral valve annulus area change ratio of 56.2% or more has 86% sensitivity and 78% specificity for predicting postoperative functional mitral stenosis

Univariate and multivariate logistic regression analyses are shown in Table 6. Analysis of risk factors for FMS using a multivariable logistic regression model, including use of the full ring type prosthetic valve ring, post-operative MV annulus area, and MV annulus area change ratio between pre- and post-MV repair, showed that only MV annulus area change ratio between pre- and post-MV repair was an independent predictor of FMS (MV annulus area change ratio increase: odds ratio 1.19, 95% confidence interval 1.09–1.33, *p* < 0.0001). A ROC curve analysis demonstrated that the MV annulus area change ratio between pre- and post-MV repair had more significant discriminatory ability to predict FMS with area under the curve (AUC) of 0.88 (*p* < 0.0001) than that of postoperative MV annulus area (AUC 0.76, *p* = 0.0027) and postoperative MV annulus area/ BSA (AUC 0.76, *p* = 0.0027). It also identified that an MV annulus area change ratio of 56.2% or more had 86% sensitivity and 78% specificity for predicting FMS (Fig. 3).

**Table 6:** Univariate and multivariate logistic regression analysis for prediction of functional mitral stenosis

Variable	Univariable		Multivariable	
	OR (95% CI)	*p* value	OR (95% CI)	*p* value
Age	0.99 (0.95–1.03)	0.61		
Body surface area	0.97 (0.07–11.3)	0.98		
AF	1.39 (0.42–4.64)	0.60		
Preoperative LAD	1.06 (0.99–1.14)	0.11		
Preoperative LVEDD	1.01 (0.94–1.08)	0.87		
Preoperative LVEF	0.95 (0.87–1.05)	0.33		
Preoperative MV annulus area	1.00 (0.99–1.00)	0.46		
Aorta cross clamp time	1.01 (0.99–1.02)	0.13		
Use of full ring type prosthetic valve ring	**4.47 (1.18–16.1)**	**0.03**	1.28 (0.29–5.69)	0.74
Resection and suture of leaflet	0.4 (0.13–1.19)	0.11		
Use of chordal replacement	0.53 (0.17–1.66)	0.29		
Postoperative stroke volume	1.0 (0.97–1.03)	0.86		
Postoperative MV annulus area	**0.99 (0.98–0.99)**	**0.0003**		
MV annulus area change ratio between pre- and post-MV repair	**1.16 (1.07–1.25)**	** < 0.0001**	**1.19 (1.09–1.33)**	** < 0.0001**

Bold values indicated statistically significance (*p*-value < 0.05)

*AF* atrial fibrillation, *LAD* left atrial diameter, *LVEDD* left ventricular end-diastolic diameter, *LVEF* left ventricular ejection fraction, *BSA* body surface area, *MV* mitral valve, *OR* odds ratio, *CI* confidence interval

### Correlations between TMPG and MV annulus area parameters

**Fig. 4:**
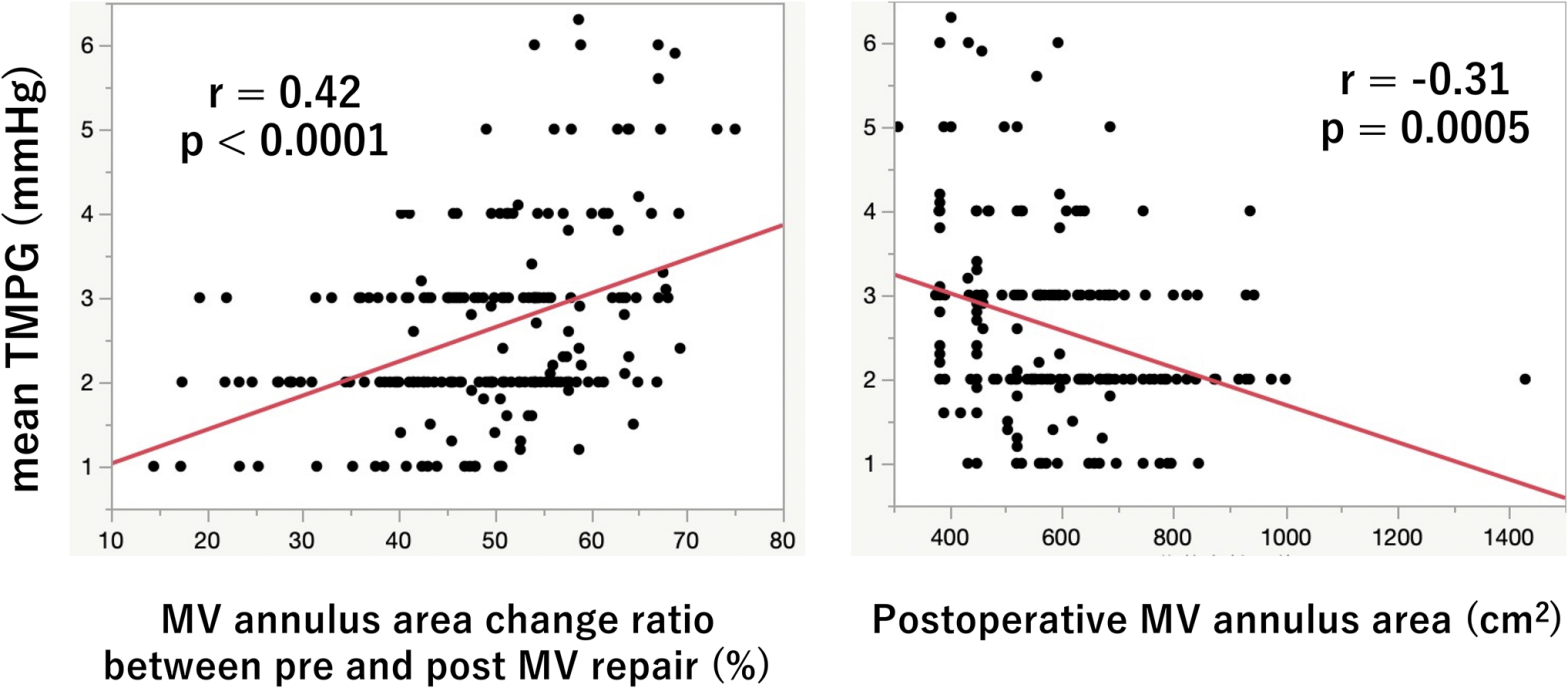
Correlations between transmitral pressure gradient and mitral valve annulus area parameters. There were significant correlations between mean transmitral pressure gradient (TMPG) and mitral valve (MV) annulus area change ratio between pre- and post-MV repair (*r* = 0.40, *p* < 0.0001) (**A**) and between mean TMPG and postoperative mitral valve area (*r* = – 0.33, *p* = 0.0005) (**B**)

There were significant correlations between mean TMPG and MV annulus area change ratio between pre- and post-MV repair (*r* = 0.40, *p* < 0.0001). This degree of correlation was similar to the correlation between TMPG and postoperative MVA (*r* = – 0.33, *p* = 0.0005) (Fig. 4).

## Discussion

In this study, 14 of 220 patients (6.4%) who underwent MV repair for DMR showed FMS. The FMS group showed a greater decrease in MV annulus area between pre- and post-MV repair than the group without FMS. MV annulus area change ratio between pre- and post-MV repair was predictive of FMS when the cut-off value was a 56.2% reduction in MV annulus area.

FMS following MV repair is known to be a poor prognostic factor after surgery, with an increased risk of postoperative congestive heart failure, atrial fibrillation, and pulmonary hypertension. [3]. In addition, FMS has been reported to cause impaired exercise tolerance, leading to decreased postoperative quality of life [9].

### The importance of conducting a high-quality MV repair

The guidelines increasingly recommend early timing of MV repair, and avoidance of both FMS and residual regurgitation is an important issue in ensuring a good prognosis and valve function after MV repair. This study has shown that the pre-and postoperative valve ring area change ratio is more influential on FMS than the postoperative MV annulus area, In addition, the preoperative MVA did not differ between FMS and non-FMS, which showed that the effect of placing a smaller ring relative to the preoperative area was most strongly associated with FMS. Pre-operative recommendations on the minimum post-operative area that should be guaranteed to avoid FMS can lead surgeons away from the selection of relatively small rings. The post-operative MVA can be estimated to some extent for each product type and size, as presented in Table 3, and the values close to the published product information have been obtained, especially for semi-rigid type full rings such as Physio2. Concerning these values and the values given in the product information, the postoperative MVA values for each prosthetic valve ring used can be estimated preoperatively. Using these values to select a size that is not less than 56% of the preoperative MVA is useful to avoid FMS. It is important to carefully evaluate preoperative 3DTEE measurements and develop an appropriate surgical strategy.

### Selection of prosthetic valve ring in MV repair

There are two factors to consider when selecting prosthetic valve ring size, avoidance of FMS and control of regurgitation. While a small prosthetic valve ring size has been reported as a risk factor for FMS [4], prosthetic valve ring size has not been reported to contribute to regurgitation control [10]. Accordingly, the main consideration when selecting prosthetic valve ring size should be the prevention of FMS. The improper treatment of valve leaflets, such as excessive leaflet resection or inappropriate artificial chordae implantation, results in short coaptation of leaflets. This in turn indicates the need to use a smaller-sized prosthetic valve ring to constrict the annulus for good leaflet coaptation. However, this increases the risk of FMS. A strategy based on the idea of repairing the valve leaflets to a form that can maintain sufficient coaptation even without the presence of a prosthetic valve ring and the use of a prosthetic valve ring to prevent future enlargement of the mitral valve annulus will allow the use of larger-sized valve rings.

### Risk factors of postoperative TMPG elevation

Various factors have been reported regarding the risk of postoperative TMPG elevation, which can be divided into valve leaflet factors and valve annulus factors. The valve leaflet factor, including leaflet resection [11, 12] or the edge-to-edge technique [13], defines the postoperative MV orifice area. On the other hand, the valve annulus factors, including a small size ring [14] or full ring type [4] prosthetic valve ring, define the postoperative MV annulus area. Recent reports note that the valve annulus factors are more substantial contributors to FMS than the valve leaflet factors [4, 9, 14]. This may be due to recent changes in MV repair techniques. In the early days of MV repair (so-called ‘French correction’), the mainstay of the technique was to resect a large portion of the prolapse lesion for morphological repair of the mitral valve. In recent years, in contrast, the aim of the technique (so-called ‘American correction’) has been to repair the mitral valve functionally, with minimum leaflet resection and an artificial tendon cord [15]. The resulting reduction in leaflet area has less effect on MV orifice area, while the influence of prosthetic valve ring morphology on postoperative hemodynamics is thought to have increased.

### Value of prosthetic valve ‘Ring Size’

It has been reported that small ring size contributes to FMS [4, 13]. These studies examined the size of the prosthetic valve ring using a ‘ring size’ specified for each product. This ‘ring size’ is defined differently for each product, however, and the area of the prosthetic valve ring differs for each product, even if the ‘ring size’ is the same. Multiple types of rings were used within these previous studies, and ‘ring size’ is hardly a uniform parameter of postoperative MV annulus size.

### The usefulness of MV annulus area in MV repair

This study demonstrated a significant correlation between postoperative MV annular area and postoperative TMPG. Using MV annulus area rather than the conventional ‘ring size’ provides more universal evidence that a smaller prosthetic ring contributes to an increase in postoperative TMPG [3, 4].

A significant correlation was also shown between postoperative TMPG and the MV annulus area change ratio between pre- and post-MV repair. This suggests that preoperative evaluation of the MV annulus area on TEE may help lead to the appropriate postoperative valve ring area to avoid postoperative FMS. Determining the prosthetic valve ring size based on this case-specific value would allow for theory-based MV repair and could lead to good and durable repair, as follows:

1. From our study, it was revealed that the pre-and postoperative mitral valve annulus area change ratio influences postoperative pressure differences and could be a predictive factor for FMS, with an optimal threshold of 56.2%.
2. Since the postoperative mitral valve annular area is determined by the prosthetic valve ring during surgery, to avoid FMS it is advisable to use the preoperative valve area as a reference and refrain from selecting an overly small prosthetic valve ring.

### Effect of using a full ring type prosthetic valve ring on MVP

In the present study, univariate analysis revealed that the use of a full ring type prosthetic valve ring was also a significant factor associated with FMS. The relationship between the use of a full ring type prosthetic valve ring and increased mitral pressure gradient has been reported in the past, especially about small ring sizes of 30 mm or less [4, 14]. Our present results also showed that the postoperative MV annulus area was significantly smaller in the FMS group, indicating that the use of small-size rings affected FMS. This is consistent with previous reports that the use of a full ring type and small-size rings affected increased TMPG. In contrast, the use of a full ring type prosthetic valve ring has been reported to reduce the risk of postoperative MR recurrence in MVP of anterior leaflet lesions compared to partial ring prostheses [9], and there are cases in which aggressive use of a full ring type prosthetic valve ring is considered in some lesions. Because the area of the full-ring prosthesis is defined for each product, the postoperative area of the full-ring prosthesis is easier to estimate preoperatively than that of a partial-ring prosthesis. Therefore, in cases where a full ring is used, the preoperative MV area can be used to calculate the postoperative valve ring area needed to avoid FMS, allowing for a preoperative strategy to determine the optimal prosthetic valve ring size for each case.

### Limitations

There are several limitations in this study. First, the study was conducted under a retrospective design with a relatively small number of patients, and the power of all statistical analyses was insufficient. Second, MV repair in this study was conducted by several surgeons, which likely resulted in differences in technique in aspects other than prosthetic valve ring selection. Lastly, there were two groups of patients whose postoperative MV images were obtained using before-discharge TEE and intraoperative TEE. Images acquired from before-discharge TEE better assess MV annulus morphology under physiologically hemodynamic conditions than those acquired from intraoperative TEE. In cases evaluated with intraoperative TEE images, we attempted to obtain images in a maximally physiologically hemodynamic state wherever possible after cardiopulmonary withdrawal. The intraclass correlation coefficient (ICC) of the postoperative MV annulus area showed an excellent correlation (ICC 0.98) in 10 patients who underwent both before-discharge and intraoperative TEE, suggesting that the use of either image had little effect on the results.

## Conclusion

Preoperative MV annulus area on TEE can be used to determine the postoperative MV annulus area to avoid FMS after MV repair.

## Data Availability

Because of the sensitive nature of the data collected for this study, the data will not be made publicly available.
